# A Multilevel Study of Alcohol Consumption in Young Adults: Self-Efficacy, Peers’ Motivations and Protective Strategies

**DOI:** 10.3390/ijerph16162827

**Published:** 2019-08-08

**Authors:** Carmen Tabernero, Bárbara Luque, Esther Cuadrado

**Affiliations:** 1 Department of Social Psychology, Instituto de Neurociencias de Castilla y León—INCYL, University of Salamanca, 37007 Salamanca, Spain; 2 Department of Psychology, Instituto Maimónides de Investigación Biomédica—IMIBIC, University of Córdoba, 14079 Córdoba, Spain

**Keywords:** drinking refusal self-efficacy, protective behavioral strategies, enhancement motivation, alcohol consumption, multilevel

## Abstract

In both developing and underdeveloped countries there has been a worrying increase in the number of young people drinking alcohol; this public health problem warrants more research. This multilevel study analyzed the influence of drinking refusal self-efficacy, peers’ motivation, and protective behavioral strategies as predictors of alcohol consumption in a sample of 261 young people arranged into 52 social groups (peers who regularly shared leisure activities). A series of questionnaires were administered individually to evaluate beliefs and behaviors related to alcohol consumption at both individual level (drinking refusal self-efficacy) and peer level (enhancement motivation and protective behavioral strategies). The results showed that the individual variable (drinking refusal self-efficacy) predicted alcohol consumption behaviors. The multilevel design allowed us to evaluate the direct and moderated effects of peers’ enhancement motivation and protective behavioral strategies on the relationship between self-efficacy and drinking behavior. These results show the importance of developing cognitive, behavioral, and educational intervention programs to increase young people and university students’ confidence and ability to use protective strategies, in order to reduce alcohol use.

## 1. Introduction

Drinking alcohol is an accepted social and cultural habit in most Western countries. The Report on Alcohol in the Spanish National Plan on Drugs [1] noted that alcohol consumption is confined to the adult population. In recent years, people start drinking at younger ages (by age 14 years most students have already tried alcohol at least once and regular consumption of alcohol begins on average at 15.1 years). The percentage of young people and teenagers drinking alcohol has increased (75% of 16-year-old students admit to having drunk alcohol in the last month, this figure exceeds 80% in 17- and 18-year-olds). The World Health Organization [2] has underlined the consequences of binge drinking (defined as 60 or more grams of pure alcohol on at least one occasion at least once per month) at an early age and expressed concern about drinking patterns. Binge drinking is related to a range of problems, both physical and social, which include disability, violence, social maladjustment, depression, physical abuse, and conflicts [3]. The WHO [2] affirmed that alcohol consumption and drinking patterns are one of the main public health problems, because of the severe health consequences; in addition, alcohol is responsible for 17.6% of all injury deaths globally. Data from the WHO [2] and qualitative studies indicate that young people’s drinking follows a harmful pattern and that there is a lack of awareness of the negative consequences of irresponsible and uncontrolled alcohol use (13.5% of all deaths among people aged 20–39 years were attributable to alcohol consumption in 2016).

These data underline the need to investigate the possible social and psychological factors related to alcohol use, as well as the strategies and possible interventions. Research has demonstrated that some psychological variables have a strong influence on alcohol consumption: drinking refusal self-efficacy is defined as a belief in one’s own capacity to refuse alcoholic beverages [4,5]; drinking motives are motivational factors that influence alcohol use [6,7]; and protective behavioral strategies are defined as strategies used to reduce the risks and consequences of drinking alcohol [8,9]. Many studies on this topic seem to agree that the peer group has a strong influence on alcohol consumption i.e., [10,11]. Just as a low drinking refusal self-efficacy probably increases a person’s use of alcohol in social contexts [12], other authors [13] have shown that, at the group level, restrictive peer norms for alcohol consumption are associated with lower levels of consumption. What contributes to creating a “culture of alcohol consumption” that encourages or discourages alcohol use and influences drinking patterns [14]. However, most studies have tried to explain individual alcohol consumption behavior without taking into account the possible effects of group pressure or the behavioral patterns of the peer group. This multilevel study was designed to fill the gap in research on group influence on alcohol use. The main aim of the study was to explore the influence of the above-mentioned motivational variables (self-efficacy, motives, and behavioral strategies) on drinking behaviors.

Self-efficacy can be defined a person’s beliefs in his or her capacity to perform target behaviors in specific situations [15]. Alcohol refusal self-efficacy can be understood as one’s belief in one’s own capacity to refuse alcoholic beverages, making it a good predictor of alcohol use [4,16,17], even during mass-attendance events [18]. Young people with low drinking refusal self-efficacy are more likely to drink alcohol and to suffer the consequences than peers with higher drinking refusal self-efficacy [19,20]. In addition, Gullo, Dawe, Kambouropoulos, Staiger, and Jackson [21] established that self-efficacy mediates the relationship between drinking expectations and alcohol consumption levels and patterns, so that self-efficacy is one of variables that influence in the adoption of protective behavioral strategies in relation to alcohol use; that is, people with high self-efficacy adopt protective behavioral strategies more frequently, and therefore would be less likely to experience the consequences of binge drinking. On the basis of the existing research we hypothesized the following.

**Hypothesis** **1:** *Individuals with lower levels of drinking refusal self-efficacy will adopt more drinking behaviors*.

Alcohol use is strongly related to motive for drinking [6,22], these motives in general are thought as proximal factors for alcohol drinking, i.e., the last factors kindling alcohol behavior in the day-to-day, yet themselves driven by more distal and general factors, such as alcohol-related positive (e.g., mood enhancement) and negative (e.g., tension reduction) expectancies [23]. Foster, Neighbors, and Prokhorov [24] claimed that there are four common motives for drinking: social (drinking as a way to celebrate and to be sociable), coping (drinking to cope with anxiety or depression), enhancement (drinking to have fun and feel good), and conformity (drinking to be liked and to fit in with a group). Young people who have more motives to drink are likely to binge drink more frequently than those that have fewer motives [25]. Among young people, the most common motives for drinking are those related to enhancement and social outcomes, which were found to be associated to binge drinking [26]. Because the enhancement motive appears to play a particularly important role in young people’s use of alcohol [26,27] we decided to focus on the relationship between enhancement motives and drinking behavior. Kuntsche and Stewart [11] noted that in social contexts individuals’ enhancement motivation was influenced by that of their peers. Moreover, because young people are particularly vulnerable to group pressure and because enhancement motives might be acquired from peers through verbal exchanges about alcohol being a good way to enhance positive states, peers’ enhancement motivation may have a strong influence on individual drinking behavior in this age group [11]. On the basis of this research we propose a second hypothesis.

**Hypothesis** **2:** *Individuals who belong to groups with higher enhancement motivation associated to alcohol consumption will adopt more drinking behaviors*.

Many studies have highlighted the importance of protective behavioral strategies that can reduce the risks and consequences of drinking alcohol [9,28]. The most common protective behavioral strategies are (a) alternating alcoholic drinks with non-alcoholic ones, (b) limiting the number of drinks one consumes, (c) assigning drivers, and (d) making sure that someone who has been drinking gets home safely [25]. Various studies have shown that young people who deploy protective behavioral strategies when drinking suffer fewer consequences than those who do not [29,30]. The latest studies show that protective behavioral strategies are directly related to lower levels of alcohol consumption and a reduction in the damage associated with alcohol use [31,32]. In the light of this research propose a third hypothesis.

**Hypothesis** **3:** *Individuals who belong to groups where use of protective behavioral strategies for limiting alcohol consumption is more common will adopt fewer drinking behaviors*.

There has been very little research on how individual and group-level characteristics interact to influence the drinking behavior of individuals. The few investigations that have carried out multilevel analyses of drinking behaviors have focused on how country-level variables (e.g., the differences regarding income inequality) influence individuals’ consumption of alcohol [33] or how specific group-level variables influence individuals’ drinking [13]. Peers have a strong influence on one another’s drinking behavior, particular in the case of young adults [34]. This makes analyses of how peer level variables interact with individual variables in relation to drinking behaviors in young adults of particular interest.

The results of the various investigations [26,35] have produced differing accounts of the influence of drinking refusal self-efficacy on teenage alcohol use. Some studies have found that drinking refusal self-efficacy is positively associated with responsible alcohol use [36], but others have not observed a direct association between the two factors [16]. This prompted us to consider what factors might moderate or mediate the relationship between drinking refusal self-efficacy and alcohol consumption. Given that young people’s behavior, and specifically their alcohol consumption behavior, is influence by their peer group [21,34,37,38], we believe that group-level variables (group enhancement motivation and group norms for use of protective behavioral strategies of this group) not only influence drinking behaviors, but also influence the relationship between individual-level variables (such as drinking refusal self-efficacy) and drinking behavior. The interaction between group-level variables (the drinking motives shared by a peer group its norms for use of protective behavioral strategies) and the individual-level variable drinking refusal self-efficacy may explain the contradictory results on the effect of drinking refusal self-efficacy on drinking behavior [16]. A group’s shared drinking motives and norms for use of protective behavioral strategies may moderate the link between individual drinking refusal self-efficacy and drinking behavior.

**Hypothesis** **4:** *Individuals with low drinking refusal self-efficacy will adopt more drinking behavior if they belong to a group where use of protective behavioral strategies is at a relatively low level*.

**Hypothesis** **5:** *Individuals with low drinking refusal self-efficacy who belong to a group with high enhancement motivation will adopt more drinking behavior if, in addition, the group norm is for a low use of protective behavioral strategies*.

**Figure 1:**
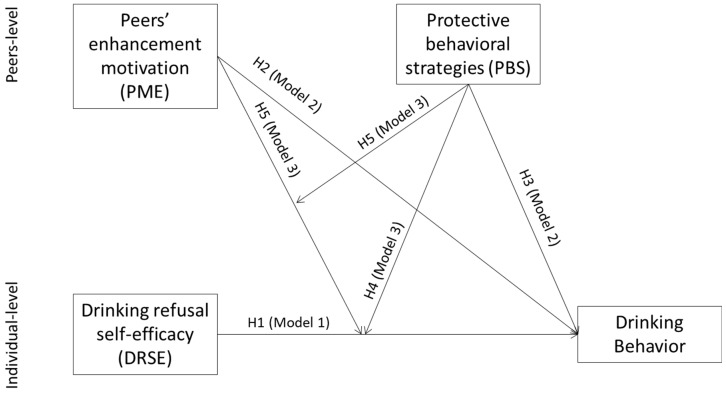
In model 1, the individual variable (drinking refusal self-efficacy, Hl) is a predictor of drinking behavior. In model 2, the collective-level variables (peers’ enhancement motivation, H2; peers’ protective behavioral strategies, H3) are considered predictors of drinking behavior. In model 3, the two way interaction peers’ protective behavioral strategies with drinking refusal self-efficacy (H4) are inserted as predictors of drinking behavior; and the three-way interaction peers’ enhancement motivation with peers’ protective behavioral strategies and drinking refusal self-efficacy (H5) are added as predictors of drinking behavior.

Our study analyzed individual (drinking refusal self-efficacy) and collective (group-level; group enhancement motivation and group norm for use of protective behavioral strategies) that influence drinking behavior in young adults who spend their leisure time with a group of peers. The specific aims were (a) to validate a multilevel model of drinking behavior incorporating factors at the individual level (drinking refusal self-efficacy) and group level (group drinking motivation and group norms for protective behavioral strategies) and (b) to identify predictors of alcohol use in young people at the individual and collective level. In order to achieve these aims, and to test the hypotheses outlined above, we first analyzed the relationship between drinking behavior and an individual-level variable, i.e., self-efficacy. Then we analyzed the relationship between drinking behavior and two collective level variables (group norm for protective behavioral strategies and group enhancement motivation). Finally, we carried out a cross-level analysis of individual variables and collective factors. Figure 1 summarizes the multilevel model and the hypotheses of the study.

## 2. Materials and Methods 

### 2.1. Participants and Procedure

All participants were volunteers. They were informed that the data would be analyzed anonymously and that they could leave the study at any time. The “Bioethical and Biosecurity Committee” of the University of Cordoba reviewed and approved the study before it began. As participants were to complete a questionnaire anonymously and there was no possibility of producing personal damage, written consent was not requested; this is in accordance with the regulations of the Spanish Ministry of Science and Innovation.

The sample comprised 261 young people (93 male; 168 female), aged 17 to 26 years (*M* = 23.53; SD = 2.81). A subsample (*n* = 52) was studying Educational Psychology at the Faculty of Educational Sciences, University of Córdoba, Spain. The rest of the participants were friends or acquaintances of these students. 

We started by recruiting Educational Psychology students, who were asked to administer the questionnaire to at least three close friends with whom they often socialized. Although other studies have considered the class as the group of influence [11], we considered that given our participants were studying at university and had a mean age of 23 years, their peer group was not their “classmates”, and so we decided the better way to collect data from groups of peers was with the collaboration of a group of reference students. They were trained to apply the questionnaires on this basis and given responsibility for recruiting the rest of the sample, and then each participant returned the questionnaire in a sealed envelope. The reference students were given a free hand to select additional participants. They provided between four and seven questionnaires each in the end. The data were organized into 52 groups; in the 52 reference students, the overwhelming majority (90.3%) consisted of five members, some of them had four members (3.85%), some had seven (3.85%) members, and a few had six members (1.92%). The majority of the sample 64.6% was unemployed, but 35.4% was engaged in some kind of paid work. Most of the participants (74%) were university students, and 8.7% were neither studying nor working. Approximately one-third of the participants (32.6%) reported that they were studying for the same degree as the reference member and a slightly higher proportion (39.8%) reported that they were studying a different subject but in the same faculty as the reference member (Faculty of Educational Sciences). Considering the sample, the differences in age, sex, employment status, and subject of study indicated a wide diversity in the sample.

To prepare them for their role in recruitment and data collection the 52 reference participants were given a brief presentation on the investigation and invited to participate in return for an extra course credit. They were told how to recruit additional participants and given instruction on completing the questionnaire and administering it. They were asked to give explained questionnaires to at least three close friends with whom they usually socialized at weekends. The questionnaire was to be completed on an individual basis. Finally, students were told that questionnaire data would be confidential, and they were informed about data protection rules. To create the aggregation for the multilevel analysis, all collected questionnaires were labeled with the number given to their reference student (from 1 to 52) and an individual identification code was created with the number of each participant in each group.

### 2.2. Measures

Sociodemographic data—age, sex, employment status, and level of education—were collected.

Drinking refusal self-efficacy questionnaire (DRSE-RA)—a revised and translated version of the DRSE-RA questionnaire [39]—was used to assess individuals’ beliefs in their ability to refuse alcohol and avoid binge drinking. Confirmatory factor analysis, in which two models were examined, was carried out to validate the questionnaire. We analyzed three primary factors, corresponding to three well-defined variables (social pressure, emotional relief, and opportunistic drinking). The questionnaire comprised 21 items describing situations and respondents used a six-point Likert scale to indicate how hard they would find it to avoid drinking in that situation: 1 = I am very sure I could NOT resist drinking; 6 = I am very sure I could resist drinking. The aforementioned factors demonstrated good reliability and validity: social pressure (α = 0.92, e.g., When I am at a party…), emotional relief (α = 0.93, e.g., When I am angry…), and opportunistic drinking (α = 0.90, e.g., When I am watching TV…). The questionnaire had good overall reliability and validity (α = 0.91).

### 2.3. Collective Variables

Enhancement drinking motivation: As enhancement drinking motivation appears to play a particularly important role in young people’s use of alcohol [27], the extent to which young people’s alcohol consumption was influenced by enhancement motivation was measured using the five enhancement factor items (e.g., Because I like the feeling) of the Modified Drinking Motive Questionnaire Revised (M-DMQ-R) [40], a questionnaire designed to measure motives for drinking. The M-DMQ-R was used in a revised, Spanish version. Participants used a six-point Likert scale ranging from 1 (almost never/never) to 6 (almost always/always) to indicate how often they drank for particular enhancement-related reasons. The reliability of the questionnaire was high (α = 0.87). Good support for aggregation was obtained (*F* (51,209) = 3.16; *p* < 0.01; ICC1 = 0.30; ICC2 = 0.68). 

Protective behavioral strategies: We assessed use of cognitive-behavioral protective strategies for avoiding or reducing alcohol consumption using a translation of the relevant seven items from the scale developed by Martens et al. [25]. The scale also contains items related to two other factors (manner of drinking strategies and strategies for reducing the negative consequences of consumption), but we did not use these because of the low reliability of these two factors the low reliability attained (original study: α = 0.74 and α = 0.59, respectively; our sample: α = 0.69 and α = 0.42, respectively). Participants indicated how often they used the seven protective behavioral strategies (e.g., Put extra ice in your drink; Leave bar or party at a predetermined time) using a six-point Likert scale ranging from 1 (almost never/never) to 6 (almost always/always). Reliability was high (α = 0.82). Exploratory factorial analysis revealed that the seven items were aggregated as a single factor that explained 47.87% of variance; all item weights were between 0.61 and 0.74. Again, good support for aggregation was obtained [*F* (51,209) = 2.75; *p* < 0.01; ICC1 = 0.26; ICC2 = 0.64]. 

Drinking behavior: The sample answered questions related to their drinking habits during the previous three weeks [39] using a calendar showing the previous three weeks, with one cell for each day. First, participants marked all the days when they had gone to party and then they marked the days on which they had consumed alcohol. The questions were “Thinking about the last three weeks, use the following calendar to indicate the days on which you went out partying (use a circle) and the days when you drunk some alcohol (use a cross).” The following questions solicited information about the number of alcoholic drinks consumed on each of the relevant days and the type of alcohol involved: “Now indicate the number of alcoholic drinks you consumed on each occasion (1 drink = 1 glass of wine, 1 can/bottle of beer or 1 single measure of spirits e.g., gin) and, finally, indicate the type of alcohol (fermented beverages, i.e., beer or wine; distilled beverages, e.g., gin) that you consumed underneath the relevant box.” To try to ensure that all participants reported their consumption using the same units of quantity we used a very explicit, easy to understand definition of one drink (see above). The number of “party days” over the three-week period ranged from 0 to 21 days (*M* = 4.45; *SD* = 3.27), whereas the number of “drinking days” ranged from 0 to 15 days (*M* = 3.67; *SD* = 2.86). The average number of drinks consumed over the three-week period ranged from 0–36 (*M* = 13.68; *SD* = 14.55) and the type of alcohol consumed varied, with fermented alcohol beverages accounting for 15.1% of drinks consumed, distilled alcohol for 31%, fermented alcohol and distilled alcohol for 44.8% (9.1% of participants consumed non-alcoholic beverages). The two variables related to alcohol consumption (number of drinking behavior days and total number of alcohol drinks) had a high correlation (*r* = 0.77; *p* < 0.001).

### 2.4. Analyses

Statistical analyzes were calculated with the SPSS program (v. 22) (IBM SPSS Statistics V22.0., SPSS Inc., Chicago, IL, USA). A multilevel model was constructed because the hypotheses referred to two levels. The influences of individual and group variables on drinking behavior were examined. Self-efficacy was measured at individual level (level 1) and enhancement motivation and protective behavioral strategies were measured at group level (level 2). Multilevel analysis is more powerful than a simple hierarchical regression analysis because it enables one to assess how individual- and group-level variables interact to explain behavior at the individual level. Moreover, multilevel models enable one to estimate between-group variance, thanks to their random component, which is not possible with simple hierarchical regression analysis. We needed to show within-group similarity variance in the group-level variables; this was done by calculating intraclass correlations. Data on the group-level variables were collected as individual-level values and then modeled at group level by aggregating the data into the 52 friendship groups. In order to assess whether the data could be aggregated in enhancement drinking motivation and PBS, *F*-test, and ICC (Intraclass Correlation index defined as the proportion of variance in the outcome explained by the grouping structure between-group variance) were calculated. According to Hox, Moerbeek, and Van de Schoot [41], to aggregate group data *F*-test must show statistical significance, and ICC values must have values higher than 0.05, 0.10, and 0.15 to consider a percentage of variance explained by the group as small, medium, or large, respectively. Moreover, in order to test the effect of the studied variables in presence of demographic factors, sex was added as dummy coded covariate at the individual level. Finally, because age is associated with alcohol consumption [2], the mean age of each group was incorporated at group level as covariate.

At all steps the parameters were measured on an individual level in order to assess unexplained variation. We started by calculating an intercept-only model (Model 0) to determine how much of the total variance in drinking behavior (Alcohol_DB) should be allocated to each level of analysis.

Model 0 equations:

Level 1:

$$Alcohol\_DB_{ij}=\beta_{0j}\;+r_{ij}\;\;$$

Level 2:

$$\beta_{0j}=\gamma_{00}+u_{0j}\;\;$$

We estimated the following three models. The first model (Model 1) measured the effect of drinking refusal self-efficacy ((DRSE) individual-level variable) on drinking behavior (at individual level) (H1) in the presence of sex as covariate.

Model 1 equations:

Level 1:

$$Alcohol\_DB_{ij}=\beta_{0j}+\beta_{1j}\times \left(SEX_{ij}\right)+\beta_{2j}\times \left(DRSE_{ij}\right)+r_{ij}\;{\;}_{\;}$$

Level 2:

$$\beta_{0j}=\gamma_{00}+\gamma_{0j}\;\;$$

$$\beta_{1j}=\gamma_{10}+u_{1j}\;\;$$

Model 2 also included estimates of the interactions between the group-level predictors: enhancement motivation (E_Motivation; H2) and protective behavioral strategies (PBS; H3)—with respect to drinking behavior. In addition, the average age of groups (Age) was added as covariate. Sex remained as covariate in the individual level.

Model 2 equations:

Level 1:

$$Alcohol\_DB_{ij}=\beta_{0j}+\beta_{1j}\times \left(SEX_{ij}\right)+\beta_{2j}\times \left(DRSE_{ij}\right)+r_{ij}$$

Level 2:

$$\beta_{0j}=\gamma_{00}+\gamma_{01}\times \left(Age_{j}\right)+\gamma_{02}\times \left(PBS_{j}\right)+\gamma_{03\;}\times \left(E\_Motivation_{j}\right)+u_{0j}$$

$$\beta_{1j}=\gamma_{10}+u_{1j}$$

$$\beta_{1j}=\gamma_{20}+u_{2j}$$

Model 3 included individual variables, group variables and cross-level, two-way and three-way interactions, in order to test the hypothesis (H4) that protective behavioral strategies moderate the association between drinking refusal self-efficacy (individual level) and drinking behavior and the hypothesis (H5) concerning the conditional effect of all the individual- and group-level predictors on drinking behavior. Age and sex were still included in order to test the effects of the variables in presence of those covariates.

Model 3 equations:

Level 1:

$$Alcohol\_DB_{ij}=\beta_{0j}+\beta_{1j}\times \left(SEX_{ij}\right)+\beta_{2j}\times \left(DRSE_{ij}\right)+r_{ij}$$

Level 2:

$$\begin{array}{ll} \beta_{0j} & =\gamma_{00}+\gamma_{01}\times \left(Age_{j}\right)+\gamma_{02}\times \left(PBS_{j}\right)+\gamma_{03\;}\times \left(E\_Motivation_{j}\right)\\ & +\gamma_{04\;}\times \left(PBS\_X\_E\_Motivation_{j}\right)+u_{0j} \end{array}$$

$$\beta_{1j}=\gamma_{10}+u_{1j}$$

$$\begin{array}{l} \beta_{2j}=\gamma_{20}+\gamma_{21}\times \left(PBSj\right)+\gamma_{22}\times \left(E\_Motivationj\right)\\ +\gamma_{23}\times \left(PBS\_X\_E\_Motivationj\right)+u_{2j} \end{array}$$

## 3. Results and Discussion

### 3.1. Preliminary Analyses

First, several ANOVAs were performed to check that the reference group of students from the Faculty of Educational Sciences (*n* = 52) was similar to the rest of the sample (*n* = 209) with respect to all the variables investigated. The results indicated that the reference group was similar to the rest of the sample with respect to all variables: drinking refusal self-efficacy (*F* (1, 259) = 0.53, ns; *M*_ref_ = 5.02, *SD* = 0.68; *M*_others_ = 4.93, *SD* = 0.79), enhancement motivation (*F* (1, 259) = 0.17, ns; *M*_ref_ = 2.85, *SD* = 1.24; *M*_others_ = 2.76, *SD* = 1.34), protective behavioral strategies (*F* (1, 259) = 0.04, ns; *M*_ref_ = 2.48, *SD* = 0.83; *M*_others_ = 2.51, *SD* = 1.19), and drinking behavior (*F* (1, 259) = 1.15, ns; *M*_ref_ = 7.67, *SD* = 5.95; *M*_others_ = 9.05, *SD* = 8.74).

**Figure 2:**
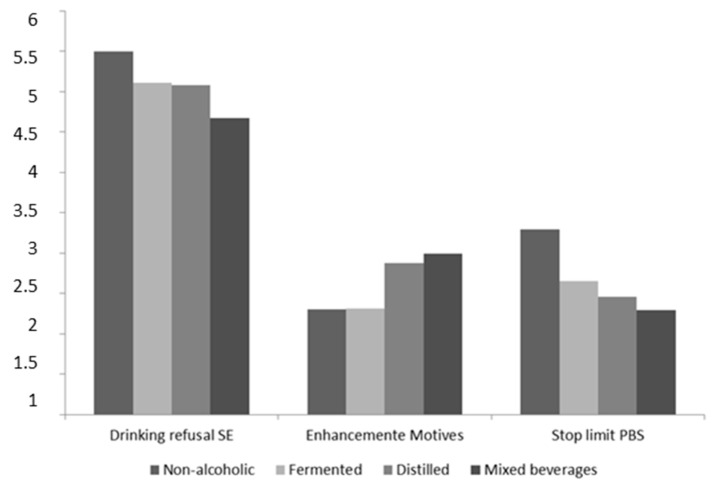
Differences in the type of beverages consumed in function of the psychosocial variables studied (drinking refusal self-efficacy, enhancement motives and protective behavioral strategies).

Second, a further series of ANOVAs with post hoc Bonferroni tests was used to relate the type of alcohol consumed to the psychosocial variables studied (see the differences on Figure 2). Students who drank a mixture of fermented and distilled drinks reported lower drinking refusal self-efficacy (*F* (3, 248) = 11.19, *p* < 0.001) than those who drank non-alcoholic beverages (*t* = 0.83, *p* < 0.001), fermented alcoholic drinks (*t* = 0.43, *p* < 0.01), and distilled alcoholic beverages (*t* = 0.40, *p* < 0.01). They also reported higher scores for enhancement motivation (*F* (3, 248) = 3.84, *p* < 0.01) than those who drank fermented beverages (*t* = 0.68, *p* < 0.05). Students who drank non-alcoholic beverages reported higher behavioral protection strategy scores (*F* (3, 248) = 6.03, *p* < 0.001) than those who drink distilled alcoholic drinks (*t* = 0.84, *p* < 0.01) or a mixture of types of alcoholic beverage (*t* = 1.01, *p* < 0.001). Age was not related to the type of alcohol consumed (*F* (3, 248) = 0.69, ns).

However, in relation with the questions associated to the alcohol consumption (number of drinking behavior days and total number of alcohol drinks), there were differences in the type of alcohol consumed. For example, students who drank a mixture of fermented and distilled drinks reported more partying days (*F* (3, 248) = 7.53, *p* < 0.001; *M* = 5.34, *SD* = 3.77) and more drinking days (*F* (3, 248) = 30.55, *p* < 0.001, *M* = 4.86, *SD* = 2.46, respectively) than those who drank non-alcoholic beverages (partying days: *t* = 4.89, *p* < 0.001, *M* = 2.65, *SD* = 2.39; drinking days: *t* = 2.68, *p* < 0.001, *M* = 0, *SD* = 0) and distilled alcoholic beverages (partying days: *t* = 1.79, *p* < 0.01, *M* = 3.55, *SD* = 2.32; drinking days: *t* = 1.96, *p* < 0.001, *M* = 2.90, *SD* = 2.21). Regarding the number of alcoholic drinks, those students who drank a mixture of fermented and distilled drinks reported higher scores (*F* (3, 248) = 21.23, *p* < 0.001; *M* = 19.88, *SD* = 16.05 and *F* (3, 248) = 20.40, *p* < 0.001, *M* = 19.84, *SD* = 16.03, respectively) than those who drink non-alcoholic beverages (*t* = 19.88, *p* < 0.001, *M* = 0, *SD* = 0; *t* = 19.84, *p* < 0.001, *M* = 0, *SD* = 0), fermented alcoholic drinks (*t* = 9.46, *p* < 0.001, *M* = 10.42, *SD* = 12.92; *t* = 9.25, *p* < 0.001, *M* = 10.59, *SD* = 13.02), and distilled alcoholic beverages (*t* = 10.33, *p* < 0.001, *M* = 9.56, *SD* = 8.21; *t* = 10.41, *p* < 0.001, *M* = 9.44, *SD* = 8.09). Consequently, there were differences in the global measure of drinking behavior: students who drank a mixture of fermented and distilled drinks had higher scores (*F* (3, 248) = 23.50, *p* < 0.001; *M* = 12.48, *SD* = 8.87) than those who drank non-alcoholic beverages (*t* =11.79, *p* < 0.001, *M* = 0.69, *SD* = 0.63), fermented alcoholic drinks (*t* = 5.17, *p* < 0.001, *M* = 7.32, *SD* = 7.46), and distilled alcoholic beverages (*t* = 6.14, *p* < 0.001, *M* = 6.34, *SD* = 4.80).

With respect to hypothesis 1, the descriptive statistics and correlations show that drinking refusal self-efficacy (*M* = 4.95; SD = 0.77) was negatively correlated with drinking behavior (*M* = 10.20; SD = 10.44; *r* = −0.33; *p* < 0.001) and enhancement motivation (*M* = 2.77; SD = 0.87) was negatively associated with use of protective behavioral strategies (*M* = 2.51; SD = 10.44; *r* = −0.18; *n.s.*).

### 3.2. Multilevel Analyses

Table 1 summarized the multilevel regression models described below:

Model 0 confirmed the existence of significant variance at group level and at individual level ($x^{2}$ = 176.38; *df* = 51; *p* < 0.001). Approximately 32% of the variance in drinking behavior depended on the peer group to which the individual belonged (ICC = 0.323).

Model 1 showed that drinking refusal self-efficacy predicted drinking behavior, confirming Hypothesis 1.

Model 2 showed that, with age as a covariate, protective behavioral strategies at group-level predicted drinking behavior, but group enhancement motivation did not. Thus, Hypothesis 2 was not supported, but Hypothesis 3 was. Moreover, it was shown that drinking refusal self-efficacy remained a predictor of drinking behavior in the presence of group-level variables, providing additional support for Hypothesis 1.

**Figure 3:**
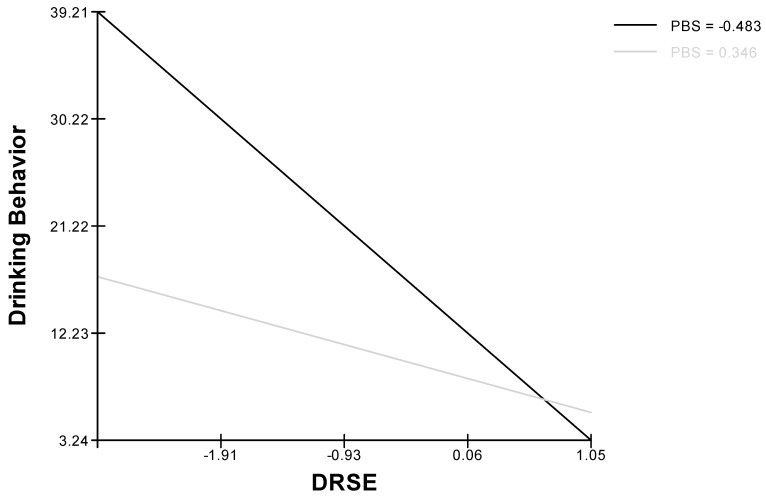
Cross-level two-way interaction effect for drinking behavior (DRSE: Drinking refusal self-efficacy; BPS: Protective behavioral strategies).

**Figure 4:**
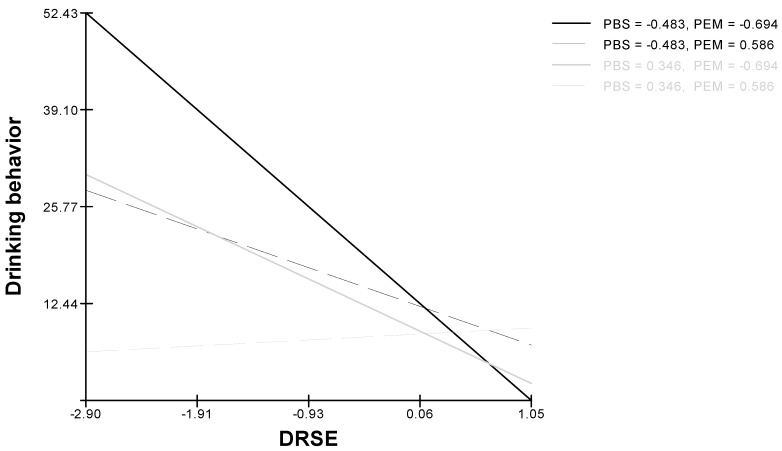
Cross-level three-way interaction effect for drinking behavior (DRSE: Drinking refusal self-efficacy; BPS: Protective behavioral strategies; PEM: Peers’ Enhancement Motivation).

Model 3 implied the reliability, with sex and age as covariates, of the two-way cross-level interaction between drinking refusal self-efficacy and protective behavioral strategies with drinking behavior as the dependent variable: protective behavioral strategies moderated the effect of self-efficacy on drinking behavior, confirming Hypothesis 4 (Figure 3). Moreover, this model demonstrated (again with sex and age as covariates) a three-way interaction between drinking refusal self-efficacy, protective behavioral strategies and peers’ enhancement motivation with drinking behavior as the dependent variable (Figure 4). Thus, Hypothesis 5 was supported.

## 4. Discussion

In this study we used a multilevel model to analyze the influence of individual variables (drinking refusal self-efficacy) and peer variables (enhancement motivation and protective behavioral strategies) on drinking behavior. The results provide us with valuable information about moderation of alcohol-related behaviors by peer-level variables. First, we offer a detailed account of the multilevel mechanisms by which drinking refusal self-efficacy (at the individual level) and enhancement motivation and protective behavioral strategies (at group level) affected individuals’ drinking behavior; all the expected relationships and interactions that were confirmed have shown to be maintained in the presence of demographic variables: sex did not predict drinking behavior, but age did. Second, our study revealed that the protective behavioral strategies (a) predicted individual drinking behavior and (b) moderated the relationship between drinking refusal self-efficacy and drinking behavior. Third, our study demonstrated that enhancement motivation moderated the effect of group norms for protective behavioral strategies on the relationship between drinking refusal self-efficacy and drinking behavior.

Like previous researchers [16,19,20,42], we found that individuals who perceived that their ability to abstain from drinking was low, also reported more elevated alcohol consumption behaviors. We showed that young people with high drinking refusal self-efficacy drank less alcohol than age peers with lower drinking refusal self-efficacy and hence, we agree with other authors [24] that confidence in one’s ability to refuse alcohol should be central to the design of future interventions in this area. At the practical level, we must include drinking refusal self-efficacy in programs targeting alcohol use in young people [43,44], because experts have consistently related drinking refusal self-efficacy to alcohol use [16]. Furthermore, some programs have been designed to improve drinking refusal self-efficacy by encouraging activities that help young people to feel better without consuming alcohol or other psychotropic substances [45].

We also examined group-level enhancement motivation and group norms for protective behavioral strategies as predictors of drinking behavior, but our data suggest that only protective behavioral strategies influenced individuals’ drinking behavior. We found that individuals in groups where use of behavioral strategies to avoid or limit drinking was common exhibited less risky drinking behavior than those whose peer group tended not to use protective behavioral strategies. In other words, young people’s drinking behavior seems to be influenced by peer pressure, in the form of group norms for use of strategies to limit or avoid alcohol consumption. This implies that programs designed to reduce or prevent alcohol consumption in young people and at-risk individuals should pay special attention to promoting the use of protective behavioral strategies within groups of peers.

Regarding the cross-level interaction posited in Hypothesis 4, our results showed that drinking refusal self-efficacy was negatively related to alcohol consumption and that this association was stronger in individuals whose peer group made frequent use of protective behavioral strategies. Moreover, the group norm for use of protective behavioral strategies had a greater effect on individual members’ alcohol consumption in the case of people with low drinking refusal self-efficacy. The drinking behavior of individuals with high drinking refusal self-efficacy level was not affected by whether they belonged to a peer group with a high or low level of use of protective behavioral strategies. Moreover, the results showed that when protective behavioral strategies are higher, both individuals with low self-efficacy and individual with high self-efficacy drink less. In the same way, previous studies have shown that individuals with low drinking refusal self-efficacy who frequently used protective behavioral strategies drank nearly seven times less than those that did not use protective behavioral strategies [46]. Ehret, Ghaidarov, and LaBrie, [47] found that in young people with low drinking refusal self-efficacy, the use of protective behavioral strategies makes an important contribution to reducing alcohol consumption and our results confirm the importance of protective behavioral strategies at the peer level: young people with low drinking refusal self-efficacy drank less if their peer group made extensive use of strategies for avoiding or limiting alcohol consumption.

Finally, in relation to the cross-level interaction posited by Hypothesis 5, our results suggest that in order to reduce their alcohol consumption, individuals with low drinking refusal self-efficacy that belong to groups with a high level of enhancement motivation for drinking need to have peers that make frequent use of protective behavioral strategies. In contrast individuals with high drinking refusal self-efficacy who were part of a peer group with a high level of enhancement motivation were not affected by their peers’ use of protective behavioral strategies. Our findings suggest that peers’ use of protective behavioral strategies has more effect on individuals’ drinking behavior than peers’ level of enhancement motivation. Once again, the role of protective behavioral strategies in programs to reduce alcohol consumption among young people has been highlighted. The seriousness of the issue of young people’s drinking means that there is real need for research that can identify possible protective strategies. The spread of intervention programs that include protective behavioral strategies would decrease the negative impact of alcohol consumption on young people [30,48].

The main limitations of the study are the sample size, the gender distribution and the data collection by the peers, so our results must be interpreted with caution and should be replicated in a larger sample to improve the analysis’ power. Moreover, because the study is cross-sectional, temporal relationships between exposure and outcome variables cannot be established. Then, further researches should replicate the results in longitudinal studies. It is also necessary to take into account the peculiarity of our study, which analyzes the effect of peers’ variables on alcohol consumption, making impossible a randomly selected sample (the groups must be a group of friends). Therefore, some unmeasured or incompletely adjusted confounders may have biased estimates of association, so our results must be interpreted with caution and should be replicated in studies in which perhaps data should be collected more naturalistically, in places where young people spend their free time together, allowing the randomization of the sample. Another limitation could be the methodology used. In this sense, to remember how many drinks individuals have consumed each day over a 3-week period would be difficult, and this difficulty could have biased the participants’ responses. Maybe in further researches it could be interesting to ask participants to register their drinking pattern for three consecutive weeks. There is also a need for more research analyzing the relationship between the type of alcohol consumed and the pattern of alcohol consumption. Our preliminary analysis showed that participants that drank a mixture of fermented and distilled alcoholic beverages had lower drinking refusal self-efficacy, higher enhancement motivation, and made less use of protective behavioral strategies than age peers with higher drinking refusal self-efficacy. Therefore, the intervention programs to prevent negative consequences of drinking behavior could be centered to give more information about alcohol types, quantity, and consequences, and even skills training at individual- and collective-level, to generate more confidence to refuse alcohol drinking.

Although there have been multiple investigations of protective behavioral strategies and alcohol use in young people [9], it is necessary to continue investigating these strategies and to incorporate them into intervention programs, as such programs have already produced good results. We treated use of protective behavioral strategies as a group-level variable; previous studies have analyzed this variable as an individual variable, although the behavioral strategies of adolescents and young people are, in general, strongly influenced by their peers. This is why we were interested in how an individual’s drinking behavior is influenced by the protective strategies adopted by his or her peer group. Our multilevel analysis fills a gap in the literature, namely how protective behavioral strategies influences drinking at the collective level.

## 5. Conclusions

In conclusion, our results suggest that interventions to reduce drinking in young adults should be based around improving individuals’ drinking refusal self-efficacy and promoting the use of protective behavioral strategies at peer group level [30]. Low drinking refusal self-efficacy levels and membership of a group that makes limited use of protective behavioral strategies can be regarded as predictors of problematic drinking behavior. Prevention and reduction interventions for alcohol consumption are already being carried out, and one of the main components of these programs is teaching young people how to say no, even when this means resisting the influence of their peers. However, Toumbourou et al. [49] claimed that such programs are not enough to tackle problematic drinking among young people. Some authors [50,51] have argued that programs designed to improve social (peers’ protective behavioral strategies) and personal (self-efficacy) competences could be more effective to reduce the consumption than those focused exclusively on preventing alcohol use. Interventions intended to improve self-efficacy reduce alcohol consumption [50]. Various studies have shown that young people with a more negative self-identity drink more than young people with a positive self-identity and that drinking in order to enhance one’s experience is related to irresponsible alcohol consumption [27]. Based on the extant evidence, including the results of our study, interventions should focus on (a) improving drinking refusal self-efficacy through activities that provide positive feelings and do not involve binge drinking [45], (b) reducing enhancement motives in the target’s peer group by encouraging participation in activities that make peers feel good without drinking alcohol [22], and (c) encouraging peer groups to use behavioral strategies to avoid or limit alcohol consumption so that members who choose to abstain from drinking or drinking less alcohol than others may also be accepted by the group [28,52].

## Figures and Tables

**Table 1:** Results of the multilevel regression predicting individual lifestyle behaviors with alcohol consumption.

Variables Included in Models	Model 0	Model 1	Model 2	Model 3
Fixed effects (with robust standard errors)				
Intercept	10.064 ***	10.249 ***	10.362 ***	10.357 ***
Self-efficacy (SE)		−5.610 ***	−5.430 ***	−5.479 ***
Sex		−2.472 (ns)	−2.459 (ns)	−2.278 (ns)
Peers’ Motives enhancement (PME)			0.475 (ns)	−0.700(ns)
Peers’ Protective Behaviors Strategies (PBS)			−2.571 ***	−5.060 **
Age			0.231 (ns)	0.228 (ns)
SE × BS				7.503 *
SE × PME				6.327 *
SE × PME × PBS				−2.257 *
Variance components (Random effects)				
Within individuals, σ^2^	73.47	35.97	36.11	36.10
Intercept, t	35.04	35.48	29.37	28.72
x^2^	176.37 ***	160.98 ***	132.94 ***	126.60
d.f.	51	43	40	39
Deviance				
(−2 × log likelihood)	1922.85	1809.76	1795.48	1781. 95
Estimated Parameters	2	7	7	7
Pseudo R2 (% explained compared to Intercept Only Model)				
	-	51.04%	50.85%	50.86%

* *p* < 0.05; ** *p* < 0.01; *** *p* < 0.001; ns = nonsignificant.

## References

[1] The Spanish Drugs Observatory Report 2017 and Statistics OEDT. http://www.pnsd.mscbs.gob.es/profesionales/sistemasInformacion/informesEstadisticas/pdf/2017_Informe_Resumen_ejecutivo_Ingles.pdf.

[2] WHO (2018). Global Status Report on Alcohol and Health 2018.

[3] Palmer R.S., Corbin W.R., Cronce J.M. (2010). Protective strategies: A mediator of risk associated with age of drinking onset. Addict. Behav..

[4] Morawska A., Oei T. (2005). Binge drinking in university students: A test of the cognitive model. Addict. Behav..

[5] Patton K., Connor J.P., Rundle-Thiele S., Dietrich T., Young R.M., Gullo M.J. (2018). Measuring adolescent drinking-refusal self-efficacy: Development and validation of the Drinking Refusal Self-Efficacy Questionnaire-Shortened Adolescent version (DRSEQ-SRA). Addict. Behav..

[6] Kuntsche E., Knibbe R.A., Engels R.C., Gmel G. (2010). Being drunk to have fun or to forget problems? Identifying enhancement and coping drinkers among risky drinking adolescents. Eur. J. Psychol. Assess..

[7] Windle R.C., Windle M. (2018). Adolescent precursors of young adult drinking motives. Addict. Behav..

[8] Dekker M.R., Jongenelis M.I., Wakefield M., Kypri K., Hasking P., Pettigrew S. (2018). A longitudinal examination of protective behavioral strategies and alcohol consumption among adult drinkers. Addict. Behav..

[9] Pearson M.R. (2013). Use of Alcohol Protective Behavioral Strategies among College Students: A Critical Review. Clin. Psychol. Rev..

[10] Edwards A.C., Maesr H.H., Prescott C.A., Kendler K.S. (2015). Multiple mechanisms influencing the relationship between alcohol consumption and peer alcohol use. Alcohol. Clin. Exp. Res..

[11] Kuntsche E., Stewart S.H. (2009). Why My Classmates Drink: Drinking Motives of Classroom Peers as Predictors of Individual Drinking Motives and Alcohol Use in Adolescence—A Mediational Model. J. Health Psychol..

[12] Haller M., Handley E., Chassin L., Bountress K. (2010). Developmental cascades: Linking adolescent substance use, affiliation with substance use promoting peers, and academic achievement to adult substance use disorders. Dev. Psychopathol..

[13] Barrientos-Gutierrez T., Gimeno D., Mangione T.W., Harrist R.B., Amick B.C. (2007). Drinking social norms and drinking behaviours: A multilevel analysis of 137 workgroups in 16 worksites. Occup. Environ. Med..

[14] Bräker A.B., Soellner R. (2017). Is Drinking Contagious? An Analysis of the Collectivity of Drinking Behavior Theory within a Multilevel Framework. Alcohol Alcohol..

[15] Bandura A., Norman P., Abraham C., Conner M. (2000). Health promotion from the perspective of social cognitive theory. Understanding and Changing Health Behaviour: From Health Beliefs to Self-Regulation.

[16] Connor J., George S., Gullo M., Kelly A., Young R. (2011). A Prospective Study of Alcohol Expectancies and Self-Efficacy as Predictors of Young Adolescent Alcohol Misuse. Alcohol Alcohol..

[17] Foster D.W., Neighbors C., Young C.M. (2014). Drink refusal self-efficacy and implicit drinking identity: An evaluation of moderators of the relationship between self-awareness and drinking behavior. Addict. Behav..

[18] Jongenelis M.I., Pettigrew S., Biagioni N. (2018). Drinking refusal self-efficacy and intended alcohol consumption during a mass-attended youth event. Subst. Use Misuse.

[19] Chassin L., Mann L.M., Sher K.J. (1988). Self-awareness theory, family history of alcoholism, and adolescent alcohol involvement. J. Abnorm. Psychol..

[20] Wiers R., Engels R.C.M.E., Lemmers L., Overbeek G. (2005). Drinking Motives, Alcohol Expectancies, Self-Efficacy, and Drinking Patterns. J. Drug Educ..

[21] Gullo M.J., Dawe S., Kambouropoulos N., Staiger P.K., Jackson C.J. (2010). Alcohol Expectancies and Drinking Refusal Self-Efficacy Mediate the Association of Impulsivity with Alcohol Misuse. Alcohol. Clin. Exp. Res..

[22] Doumas D.M., Miller R., Esp S. (2017). Impulsive sensation seeking, binge drinking, and alcohol-related consequences: Do protective behavioral strategies help high risk adolescents?. Addict. Behav..

[23] Studer J., Baggio S., Dupuis M., Mohler-Kuo M., Daeppen J.-B., Gmel G. (2016). Drinking Motives As Mediators of the Associations between Reinforcement Sensitivity and Alcohol Misuse and Problems. Front. Psychol..

[24] Foster D.W., Neighbors C., Prokhorov A. (2014). Drinking motives as moderators of the effect of ambivalence on drinking and alcohol-related problems. Addict. Behav..

[25] Martens M.P., Pederson E.R., Labrie J.W., Ferrier A.G., Cimini M.D. (2007). Measuring alcohol-related protective behavioral strategies among college students: Further examination of the Protective Behavioral Strategies Scale. Psychol. Addict. Behav..

[26] Labrie J., Pedersen E.R., Neighbors C., Hummer J.F. (2008). The Role of Self-Consciousness in the Experience of Alcohol-Related Consequences among College Students. Addict. Behav..

[27] Leigh J., Neighbors C. (2009). Enhancement Motives Mediate the Positive Association between Mind/Body Awareness and College Student Drinking. J. Soc. Clin. Psychol..

[28] Grazioli V.S., Dillworth T., Witkiewitz K., Andersson C., Kilmer J.R., Pace T., Fossos-Wong N., Carroll H., Berglund M., Daeppen J.-B. (2015). Protective behavioral strategies and future drinking behaviors: Effect of drinking intentions. Psychol. Addict. Behav..

[29] Kulesza M., Apperson M., Larimer M.E., Copeland A.L. (2010). Brief alcohol intervention for college drinkers: How brief is?. Addict. Behav..

[30] Martens M.P., Martin J.L., Littlefield A.K., Murphy J.G., Cimini M.D. (2011). Changes in Protective Behavioral Strategies and Alcohol Use among College Students. Drug Alcohol Depend..

[31] Borden L.A., Martens M.P., McBride M.A., Sheline K.T., Bloch K.K., Dude K. (2011). The role of college students’ use of protective behavioral strategies in the relation between binge drinking and alcohol-related problems. Psychol. Addict. Behav..

[32] Kenney S.R., Labrie J.W. (2013). Use of Protective Behavioral Strategies and Reduced Alcohol Risk: Examining the Moderating Effects of Mental Health, Gender and Race. Psychol. Addict. Behav..

[33] Parry-Langdon N., Boyce W., Elgar F.J., Roberts C. (2005). Income inequality and alcohol use: A multilevel analysis of drinking and drunkenness in adolescents in 34 countries. Eur. J. Public Health.

[34] Bot S.M., Engels R.C.M.E., Knibbe R.A. (2005). The effects of alcohol expectancies on drinking behaviour in peer groups: Observations in a naturalistic setting. Addiction.

[35] Park A., Sher K.J., Krull J.L. (2006). Individual differences in the “Greek effect” on risky drinking: The role of self-consciousness. Psychol. Addict. Behav..

[36] Rabaglietti E., Burk W.J., Giletta M. (2012). Regulatory self-efficacy as a moderator of peer socialization relating to Italian adolescents’ alcohol intoxication. Soc. Dev..

[37] Knecht A.B., Burk W.J., Weesie J., Steglich C. (2011). Friendship and alcohol use in early adolescence: A multilevel social network approach. J. Res. Adolesc..

[38] Osgood D.W., Ragan D.T., Wallace L., Gest S.D., Feinberg M.E., Moody J. (2013). Peers and the Emergence of Alcohol Use: Influence and Selection Processes in Adolescent Friendship Networks. J. Res. Adolesc..

[39] Young R.M., Hasking P.A., Oei T.P., Loveday W. (2007). Validation of the Drinking Refusal Self-Efficacy Questionnaire—Revised in an Adolescent Sample (DRSEQ-RA). Addict. Behav..

[40] Grant V.V., Stewart S.H., O’Connor R.M., Blackwell E., Conrod P.J. (2007). Psychometric evaluation of the five-factor Modified Drinking Motives Questionnaire—Revised in undergraduates. Addict. Behav..

[41] Hox J.J., Moerbeek M., Van de Schoot R. (2017). Multilevel Analysis: Encyclopedia of Social Measurements.

[42] Oei T.P., Jardim C.L. (2007). Alcohol expectancies, drinking refusal self-efficacy and drinking behaviour in Asian and Australian students. Drug Alcohol Depend..

[43] Choi H.J., Hecht M., Smith R.A. (2017). Investigating the Potential Impact of Social Talk on Prevention Through Social Networks: The Relationships Between Social Talk and Refusal Self-Efficacy and Norms. Prev. Sci..

[44] Collins S.E., Witkiewitz K., Larimer M.E. (2011). The Theory of Planned Behavior as a Predictor of Growth in Risky College Drinking. J. Stud. Alcohol Drugs.

[45] Palfai T.P., Ralston T.E., Wright L.L. (2011). Understanding university student drinking in the context of life goal pursuits: The meditational role of enhancement motives. Personal. Individ. Differ..

[46] LaBrie J.W., Lac A., Kenney S.R., Mirza T. (2011). Protective behavioral strategies mediate the effect of drinking motives on alcohol use among heavy drinking college students: Gender and race differences. Addict. Behav..

[47] Ehret P.J., Ghaidarov T.M., Labrie J.W. (2013). Can you say no? Examining the relationship between drinking refusal self-efficacy and protective behavioral strategy use on alcohol outcomes. Addict. Behav..

[48] Kenney S.R., Napper L.E., Labrie J.W., Martens M.P. (2014). Examining the Efficacy of a Brief Group Protective Behavioral Strategies Skills Training Alcohol Intervention with College Women. Psychol. Addict. Behav..

[49] Toumbourou J., Stockwell T., Neighbors C., Sturge J., Rehm J., Marlatt G.A. (2007). Interventions to reduce harm associated with adolescent substance use. Lancet.

[50] Carver C.S., Scheier M.F. (1999). Themes and issues in the self-regulation of behavior. Adv. Soc. Cogn..

[51] Foster D.W., Young N., Neighbors C. (2014). I think I can’t: Drink refusal self-efficacy as a mediator of the relationship between self-reported drinking identity and alcohol use. Addict. Behav..

[52] DeMartini K.S., Palmer R.S., Leeman R.F., Corbin W.R., Toll B.A., Fucito L.M., O’Malley S.S. (2013). Drinking less and drinking smarter: Direct and indirect protective strategies in young adults. Psychol. Addict. Behav..

